# The association between medication use and health-related quality of life in multimorbid older patients with polypharmacy

**DOI:** 10.1007/s41999-024-01036-4

**Published:** 2024-08-20

**Authors:** Charlotte Falke, Fatma Karapinar, Marcel Bouvy, Mariëlle Emmelot, Svetlana Belitser, Benoit Boland, Denis O’Mahony, Kevin D. Murphy, Moa Haller, Paola Salari, Matthias Schwenkglenks, Nicolas Rodondi, Toine Egberts, Wilma Knol

**Affiliations:** 1 Division of Pharmacoepidemiology and Clinical Pharmacology, Faculty of Science, Utrecht Institute for Pharmaceutical Sciences, Utrecht University, Utrecht, the Netherlands; 2 Department of Clinical Pharmacy and Toxicology, Maastricht University Medical Center+, Maastricht, the Netherlands; 3 Department of Clinical Pharmacy, CARIM Cardiovascular Research Institute Maastricht, Maastricht University, Maastricht, the Netherlands; 4 Geriatric Medicine Department and Expertise Centre Pharmacotherapy in Old Persons, University Medical Centre Utrecht, Utrecht University, Utrecht, the Netherlands; 5 Institute of Health and Society (IRSS), Université Catholique de Louvain (UCLouvain), Louvain, Belgium; 6 Geriatric Medicine, Cliniques Universitaires Saint-Luc, avenue Hippocrate 10, 1200 Brussels, Belgium; 7 Department of Medicine, University College Cork, Cork, Ireland; 8 Department of Geriatric and Stroke Medicine, Cork University Hospital, Cork, Ireland; 9 School of Pharmacy, University College Cork, Cork, Ireland; 10 Institute of Primary Health Care (BIHAM), University of Bern, Bern, Switzerland; 11 Department of General Internal Medicine, Inselspital, Bern University Hospital, University of Bern, Bern, Switzerland; 12 Institute of Pharmaceutical Medicine (ECPM), University of Basel, Klingelbergstrasse, 61, 4056 Basel, Switzerland; 13 Joint Research Centre (JRC), European Commission, Ispra, Italy

**Keywords:** HRQoL, Quality of life, Medication use, Medication complexity, Polypharmacy

## Abstract

**Purpose:**

To explore the association between medication use-related factors and health-related quality of life (HRQoL) in older hospitalised multimorbid patients with polypharmacy.

**Methods:**

This cross-sectional study used the intervention arm data of the OPERAM trial (hospitalised patients ≥ 70 years with polypharmacy). HRQoL was assessed using the visual analogue scale (EQ-VAS) and the EQ-5D index score of the EuroQol questionnaire (EQ-5D-5L). Lower or higher EQ-VAS/EQ-5D was based on the median of the study population. Medication use-related factors included hyperpolypharmacy (≥ 10 medications), anticholinergic and sedative burden, appropriateness of medication (STOPP/START criteria), high-risk medication for hospital (re)admission, medication complexity and adherence. Multivariable logistic regression analysis was used to assess the association between medication use-related factors and HRQoL.

**Results:**

A total of 955 patients were included (mean age 79 years, 46% female, median EQ-VAS of 60, median EQ-5D of 0.60). Opioids use was associated with lower EQ-5D and EQ-VAS (aOR EQ-5D: 2.10; 95% CI 1.34–3.32, EQ-VAS: 1.59; 1.11–2.30). Hyperpolypharmacy (aOR 1.37; 1.05–1.80), antibiotics (aOR 1.64; 1.01–2.68) and high medication complexity (aOR 1.53; 1.10–2.15) were associated with lower EQ-VAS. A high anticholinergic and sedative burden (aOR 1.73; 1.11–2.69), presence of multiple prescribing omissions (aOR 1.94; 1.19–3.17) and benzodiazepine use (aOR 2.01; 1.22–3.35) were associated with lower EQ-5D. Especially in hyperpolypharmacy patients, high anticholinergic and sedative burden and medication complexity were associated with a lower HRQoL.

**Conclusion:**

Several medication use-related factors are significantly associated with a lower HRQoL in hospitalised older patients. Medication complexity is a novel factor, which should be considered when evaluating medication use of older patients with hyperpolypharmacy.

## Key summary points

**Aim:**

To explore the association between medication use-related factors and quality of life in older hospitalised patients with polypharmacy.

**Findings:**

Hyperpolypharmacy, a high anticholinergic and sedative burden, presence of multiple prescribing omissions (based on START criteria), the use of opioids, antibiotics and benzodiazepines, and high medication complexity were associated with a lower quality of life.

**Message:**

Due to the association with quality of life, evaluating medication use-related factors, especially medication complexity as a novel factor, is important for hospitalised older patients with (hyper)polypharmacy.

## Introduction

Health-related quality of life (HRQoL) is a patient-reported outcome used in clinical trials to assess individuals’ perceptions of mental and physical well-being [1]. For most older people, preserving quality of life is more important than prolonging their lifespan [2, 3]. As individuals age, they become increasingly susceptible to chronic illnesses, leading to the use of a growing number of prescribed medications. Medications can cure, reduce and prevent illnesses or symptoms [4] with the ultimate aim of contributing to quality adjusted life years. However, polypharmacy has also been associated with undesired health outcomes, such as increased morbidity and mortality, and could therefore reduce quality of life [4, 5]. In addition, age-related changes in pharmacokinetics and pharmacodynamics could increase the susceptibility of older people to negative effects of medication [1, 6–9].

Although several studies have investigated the association between characteristics of medication use, such as potentially inappropriate prescribing, adherence and number of medications, and HRQoL, the results have been inconsistent. While most studies suggest that polypharmacy may deteriorate HRQoL, a meta-analysis did not find the number of medications to be a risk factor [5, 10–14]. Additionally, to the best of our knowledge, the role of medication complexity in HRQoL has not been explored.

The older multimorbid population with polypharmacy is heterogeneous. Existing interventions primarily focus on mitigating medication-related issues, often giving less priority to quality of life, despite its importance for many older people [15, 16]. A better understanding of the impact of medication use-related factors could assist healthcare professionals in developing medication-related interventions to preserve or improve the HRQoL in this vulnerable population. Therefore, the aim of this study is to explore whether medication use-related factors are associated with health-related quality of life (HRQoL) in older hospitalised multimorbid patients with polypharmacy.

## Methods

### Setting, study population and study design

This cross-sectional study was conducted using baseline data from the OPERAM trial (Optimising thERapy to prevent Avoidable hospital admission in the Multimorbid older people), which has been described in detail elsewhere [17, 18]. In brief, the OPERAM trial was a cluster-randomized, controlled trial conducted in four European countries (Belgium, Ireland, the Netherlands and Switzerland). This trial studied the effect of a structured medication review, supported by a software-based clinical decision support system (CDSS) with integrated STOPP/START criteria, on medication-related hospital admissions within one year and other clinical outcomes like HRQoL.

Participants in OPERAM were patients aged 70 years or older with three or more chronic conditions (defined by international classification of diseases, 10th revision, codes [ICD10]) and polypharmacy (defined as five or more daily medications used). Exclusion criteria were planned transfer to palliative care within 24 h after admission, report of a structured medication review by a clinician within 2 months before enrolment and inability to provide written informed consent by the participant, or a proxy in case of cognitive impairment [17].

In the current study, only patients from the intervention group were included as only for these participants appropriateness of medication could be derived from the used software tool. Patients who had no observations on both EQ-VAS and EQ-5D at baseline were excluded, because of the complete absence of data on HRQoL.

### Study variables, data collection and classification

The data collection is described in the OPERAM trial intervention protocol [18]. The measurements of the patients on the day of admission to a hospitalisation ward (baseline) were selected. These measurements were collected before the intervention took place (see Table 1).

**Table 1:** Table of study variables and assessments

Variable (Assessment)	Hospital ward admission	
	Pre-study/screening (before inclusion)	Baseline
Quality of life (EQ-5D)		X
Medication information (SHiM): hyperpolypharmacy, anticholinergic and sedative burden, high-risk medication for hospital (re)admission, medication complexity		X^a^
Appropriateness of medication (STOPP/START)		X^b^
Medication Adherence (MMAS-8)^© c^		X^a^
Demography: age, gender, BMI, smoking status, level of education, nonindependent living	X	
Dementia		X
Renal impairment		X
DADL (Barthel index)		X
Number of falls during the past year		X^a^
Hospital specifics: trial site, ward type, type of hospital admittance	X	
Number of hospital admission during past year		X^a^

^a^Retrospective data, representing the situation prior to hospitalisation

^b^Generated at baseline and confirmed by the pharmacotherapy team prior to intervention

^c^Use of the Morisky Medication Adherence Measure questionnaire is protected by U.S. copyright laws. Permission for use is required. A license agreement was obtained from Donald E Morisky, ScD, ScM, MSPH, Professor, Department of Community Health Sciences, UCLA Fielding School of Public Health, 650 Charles E Young Drive South, Los Angeles, CA 90095–1772, USA (dmorisky@ucla.edu)

### Health-related quality of life

**Fig. 1:**
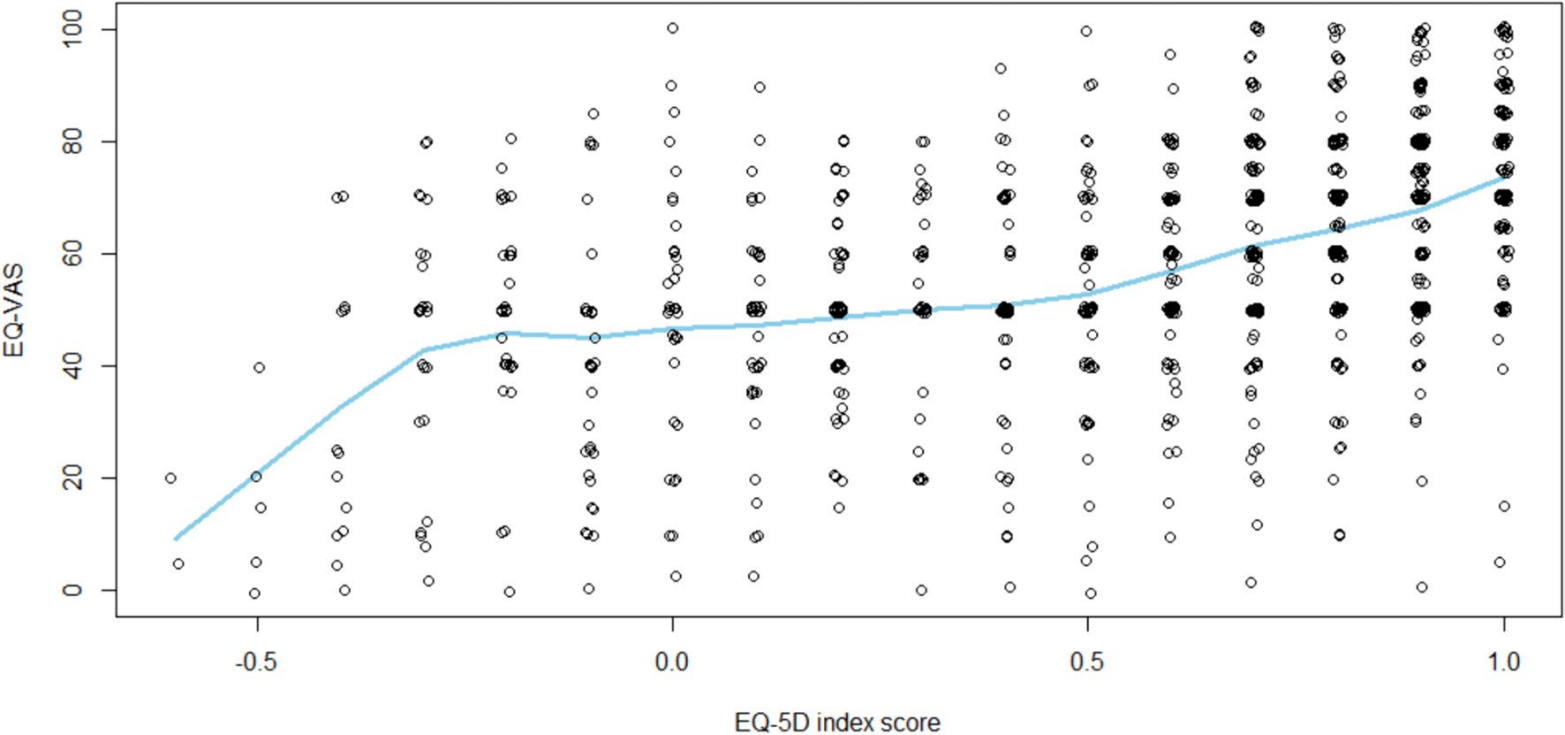
Scatter plot depicting the nonlinear association of EQ-VAS and EQ-5D index scores of the study population at baseline

The primary outcome of this study was the HRQoL, which was assessed using the EuroQol questionnaire. This instrument consists of a visual analogue scale (EQ-VAS), ranging from 0 to 100 (where 100 represents the highest conceivable health status), and an index score (EQ-5D). The index score is derived from questions on five dimensions (mobility, self-care, usual activities, pain/discomfort and anxiety/depression), which are rated on a five-point scale (0–4) and converted into the index score using a validated, country-specific scoring algorithm. Since there was no Swiss country-specific scoring algorithm available, the German algorithm was used as the closest resembling population. An index value of 1 represents ‘full health’; 0 represents ‘death’, and negative values represent ‘worse than death’ [19]. It was checked whether there was a significant linear relation between EQ-VAS and EQ-5D (see Fig. 1). As no clear linear relation was observed, EQ-VAS and EQ-5D were considered separate indicators for HRQoL. Scores below the median EQ-5D were considered indicative of lower HRQoL of the study population; the same applies to EQ-VAS. This methodology was previously used in other publications [12, 20].

### Medication use-related factors

The following medication use-related factors were evaluated: hyperpolypharmacy, anticholinergic and sedative burden, the appropriateness of medication, high-risk medication for hospital (re)admissions, medication complexity and adherence to medication. All medication information was assessed using the Structured History taking of Medications (SHiM) questionnaire [21] by the patient or proxy at baseline, and reflected the chronic medication use (> 30 days) prior to hospitalisation (see appendix Table 1):

- Hyperpolypharmacy was defined as ≥ 10 chronic medications used, including non-prescription medication.
- Anticholinergic and sedative burden was assessed using the Drug Burden Index (DBI). The DBI is an equation following DBI = Σ D/(δ + D), where D = daily dose taken for a specific anticholinergic and/or sedative medication, and δ = minimum recommended daily dose for that medication according to reference sources [22, 23]. The total DBI is the sum of the burden scores of all the anticholinergic and/or sedative medications [22, 24]. The total DBI scores were divided into three categories based on previous studies, where a DBI score ≥ 1 was considered a high anticholinergic/sedative burden, and a score of 0 meant no burden [25].
- Appropriateness of medication, defined as potential prescribing omissions and potentially inappropriate medication. These were based on CDSS-generated START and STOPP recommendations, respectively, which were confirmed as clinically relevant by the pharmacotherapy team of the OPERAM trial during hospitalisation [17, 26, 27].
- High-risk medication for hospital (re-)admissions in older patients was defined based on previous literature [8, 28, 29]. The selected high-risk medication groups were diuretics, anticoagulants, anti-arrhythmics, oral corticosteroids, antidiabetics, opioids, antibiotics, benzodiazepines, antidepressants and oral chemotherapy.
- Medication complexity was assessed using the Medication Regimen Complexity Index (MRCI) [30]. The MRCI uses three different components to quantify medication regimen complexity: pharmaceutical dosage forms (A), dosage frequencies (B) and additional instructions (C). The sum of the components’ subscores is the total MRCI score [30, 31]. The minimum score is 0, when no medication is used, and there is no upper limit as the number of medications is not limited.
- Medication adherence was self-reported and assessed using the Morisky Medication Adherence Scale-8 (MMAS-8)^©^ questionnaire at baseline, reflecting the adherence prior to hospitalisation. A score of 8 means good adherence; 6–8 is considered medium adherence, and a score lower than 6 is considered low adherence [32–34].

### Potential confounders

Sociodemographic as well as clinical characteristics could influence HRQoL, and clinical characteristics are proxies for patients’ health. The following patient characteristics were included: age, gender, body mass index (BMI), smoking status, level of education, nonindependent living, dementia, renal impairment (estimated glomerular filtration rate < 50 ml/min), dependency in activities of daily living at baseline (DADL, measured with the Barthel index), number of falls during the past year, trial site, ward type (medical or surgical), type of hospital admittance (elective/nonelective) and number of hospital admission(s) during the past year [1, 6, 7, 9, 35, 36].

### Data analysis

The data were analysed using the software program R version 4.1.2. The missing data were checked for random distribution over the defined HRQoL groups. For as-needed medication and missing doses, the median dose of the study population for that medication was used, and sensitivity analyses were performed. For medication adherence, missing values were imputed based on the available individuals’ answers. Determinants with continuous values were dichotomised or categorized into tertiles based on their distribution or on the threshold from literature (DBI, adherence, BMI and DADL) and presented as the numbers with their respective percentages within the categories. Chi-square tests were used to identify differences between the groups with lower and higher HRQoL. Univariable and multivariable logistic regression-based analyses were performed to explore the association of medication use-related factors with HRQoL. All significant potential confounders (*p* < 0.1) were considered in the multivariable stage, and stepwise backward regression models were fitted. The results of the univariable and multivariable models are presented as odds ratios (OR) or adjusted odds ratios (aOR) with their respective 95% confidence intervals (CI) and *p*-values. *p*-values < 0.05 were considered statistically significant. All variables were checked for multicollinearity in the model, with a variance inflation factor over 2.5 as a threshold [37]. Effect modification was checked by stratification, and all possible interactions were checked for significance.

## Results

### Study population

The intervention group in the OPERAM trial consisted of 963 patients. For eight patients, neither EQ-5D nor EQ-VAS data were available at baseline. The study population therefore consisted of the remaining 955 patients, who had an average age of 79 years, used a median of 10 chronic medications and of whom 46% were female (see Table 2). The median HRQoL was 60 according to the EQ-VAS and 0.60 according to the EQ-5D index score. The interquartile range for the lower EQ-VAS was 30–50, with a median of 50 and for the higher EQ-VAS this was 65–80, with a median of 70. The interquartile range for the lower EQ-5D was −0.1–0.4, with a median of 0.2 and for the higher EQ-5D this was 0.7–0.9, with a median of 0.8.

**Table 2:** Characteristics of the patients divided into groups based on HRQoL^a^

	Lower EQ-VAS	Higher EQ-VAS	*p*-value	Lower EQ-5D	Higher EQ-5D	*p*-value
Total number of patients	449	478		402	548	
Age ≥ 80 years	199 (44.3)	220 (46.0)	0.649	197 (49.0)	236 (43.1)	0.080
Female	224 (49.9)	210 (43.9)	0.080	205 (51.0)	232 (42.3)	0.010
Body mass index ≥ 30 kg/m^2^	94 (22.7)	99 (22.1)	0.909	99 (26.3)	97 (19.2)	0.016
Current smoker	46 (10.2)	30 (6.3)	0.038	43 (10.7)	32 (5.9)	0.009
Level of education			0.026			0.025
Less than high school	140 (31.5)	113 (23.8)		127 (32.0)	131 (24.1)	
High school	219 (49.3)	253 (53.3)		194 (48.9)	288 (53.0)	
University	85 (19.1)	109 (22.9)		76 (19.1)	124 (22.8)	
Nonindependent living	99 (22.0)	62 (13.0)	< 0.001	114 (28.4)	53 (9.7)	< 0.001
Dementia	37 (8.2)	25 (5.2)	0.089	31 (7.7)	34 (6.2)	0.436
Renal impairment (eGFR < 50 ml/min)	156 (35.9)	151 (32.1)	0.254	132 (34.3)	174 (32.2)	0.544
DADL (Barthel index) ^b^: Moderate or severe	269 (60.7)	164 (34.6)	< 0.001	276 (70.2)	169 (31.0)	< 0.001
≥ 1 Fall(s) during past year	99 (22.2)	77 (16.2)	0.025	103 (25.9)	77 (14.2)	< 0.001
Trial site			0.589			< 0.001
Bern	200 (44.5)	228 (47.7)		129 (32.1)	309 (56.4)	
Cork	63 (14.0)	71 (14.9)		40 (10.0)	98 (17.9)	
Louvain	71 (15.8)	74 (15.5)		68 (16.9)	80 (14.6)	
Utrecht	115 (25.6)	105 (22.0)		165 (41.0)	61 (11.1)	
Ward specialism (surgical/medical): Surgical	88 (19.6)	103 (21.5)	0.514	95 (23.6)	102 (18.6)	0.071
Type of hospital admittance: Nonelective	335 (75.3)	364 (76.3)	0.774	285 (72.0)	430 (78.5)	0.026
≥ 1 Hospital admission(s) during past year	112 (25.0)	96 (20.1)	0.090	101 (25.2)	110 (20.1)	0.075
Medication use-related factors						
Hyperpolypharmacy (≥ 10 medications)	264 (58.8)	239 (50.0)	0.009	236 (58.7)	271 (49.5)	0.006
Anticholinergic and sedative burden			0.834			< 0.001
DBI = 0	221 (49.2)	244 (51.0)		178 (44.3)	303 (55.3)	
DBI 0–1	134 (29.8)	135 (28.2)		119 (29.6)	154 (28.1)	
DBI ≥ 1	94 (20.9)	99 (20.7)		105 (26.1)	91 (16.6)	
Appropriateness of medication:						
No. of prescribing omissions			0.463			0.004
0	186 (49.5)	227 (53.8)		160 (45.5)	259 (55.7)	
1	121 (32.2)	122 (28.9)		113 (32.1)	138 (29.7)	
≥ 2	69 (18.4)	73 (17.3)		79 (22.4)	68 (14.6)	
No. of inappropriate medications			0.798			0.582
0–1	102 (27.1)	122 (28.9)		93 (26.4)	137 (29.5)	
2–4	100 (26.6)	114 (27.0)		99 (28.1)	120 (25.8)	
≥ 5	174 (46.3)	186 (44.1)		160 (45.5)	208 (44.7)	
High-risk medication^c^						
Antidiabetics	126 (28.1)	116 (24.3)	0.215	118 (29.4)	125 (22.8)	0.027
Opioids	94 (20.9)	63 (13.2)	0.002	94 (23.4)	64 (11.7)	< 0.001
Antibiotics	50 (11.1)	31 (6.5)	0.017	42 (10.4)	41 (7.5)	0.138
Benzodiazepines	60 (13.4)	48 (10.0)	0.141	60 (14.9)	50 (9.1)	0.008
Antidepressants	118 (26.3)	90 (18.8)	0.008	107 (26.6)	103 (18.8)	0.005
Medication complexity			< 0.001			< 0.001
< 16.5	130 (29.0)	169 (35.4)		114 (28.4)	203 (37.0)	
16.5–25.4	137 (30.5)	175 (36.6)		127 (31.6)	188 (34.3)	
≥ 25.5	182 (40.5)	134 (28.0)		161 (40.0)	157 (28.6)	
Medication adherence (MMAS-8)^© d^			0.930			0.139
Low adherence	64 (15.1)	73 (16.0)		65 (17.2)	74 (14.1)	
Medium adherence	173 (40.8)	183 (40.1)		139 (36.8)	225 (43.0)	
Good adherence	187 (44.1)	200 (43.9)		174 (46.0)	224 (42.8)	

Missing data: EQ-5D, 5 (0.5%); EQ-VAS, 28 (2.9%); BMI, 71 (7.4%); smoking status, 1 (0.1%); number of falls during the previous year, 8 (0.8%); level of education, 10 (1.0%); number of hospitalisations in the previous year, 2 (0.2%); admission type, 6 (0.6%); renal function, 24 (2.5%); Barthel Index of ADL, 11 (1.2%); medication adherence, 49 (5.1%); No of prescribing omissions, 133 (13.9%); No of inappropriate medications, 133 (13.9%)

^a^The values are numbers (percentages)

^b^Dependency on activities of daily living (DADL) measured with the Barthel index, a score of ≤ 60 is considered a severe dependency, 60–90 is considered moderate dependency and > 90 almost no dependency[10]

^c^Only the high-risk medication (medication with a high risk for hospital (re)admissions in patients) with significant differences in proportions are displayed

^d^Use of the Morisky Medication Adherence Measure questionnaire is protected by U.S. copyright laws. Permission for use is required. A license agreement was obtained from Donald E Morisky, ScD, ScM, MSPH, Professor, Department of Community Health Sciences, UCLA Fielding School of Public Health, 650 Charles E Young Drive South, Los Angeles, CA 90095–1772, USA (dmorisky@ucla.edu)

### Medication use-related factors

In Table 3 the associations of medication use with lower EQ-VAS and EQ-5D index scores are presented. The use of opioids showed an association with both lower EQ-VAS and EQ-5D (aOR 1.59; 95% CI 1.11–2.30 and aOR 2.1; 95% CI 1.34–3.32, respectively). Hyperpolypharmacy, use of antibiotics and higher medication complexity showed an association with EQ-VAS (aOR 1.37; 95% CI 1.05–1.80, aOR 1.64; 95% CI 1.01–2.68 and aOR 1.53; 95% CI 1.10–2.15, respectively). These factors were not significantly associated with a lower EQ-5D. For EQ-5D, an association was found between lower EQ-5D index scores and anticholinergic and sedative burden of at least 1, the presence of at least two prescribing omissions and benzodiazepines (aOR 1.73; 95% CI 1.11–2.69, aOR 1.94; 95% CI 1.19–3.17 and aOR 2.01; 95% CI 1.22–3.35, respectively). Adherence and the number of inappropriate medications showed no association with EQ-VAS and EQ-5D in this study population.

**Table 3:** Association of medication use-related factors with lower EQ-VAS and EQ-5D index scores

Medication use-related factors	crude OR (CI) EQ-VAS	aOR (CI) EQ-VAS ^a^	crude OR (CI) EQ-5D	aOR (CI) EQ-5D ^b^
Total number of patients	927	916	950	855
Hyperpolypharmacy	1.43 (1.10; 1.85)	1.37 (1.05; 1.80)	1.45 (1.12; 1.89)	1.30 (0.93; 1.84)
Anticholinergic and sedative burden				
DBI = 0	Ref	Ref	Ref	Ref
DBI 0–1	1.10 (0.81; 1.48)	1.05 (0.76; 1.43)	1.32 (0.97; 1.78)	1.11 (0.75; 1.64)
DBI ≥ 1	1.05 (0.75; 1.47)	0.88 (0.62; 1.25)	1.96 (1.40; 2.75)	1.73 (1.11; 2.69)
Appropriateness of medication				
No. of prescribing omissions				
0	Ref	Ref	Ref	Ref
1	1.21 (0.88; 1.66)	1.16 (0.83; 1.62)	1.33 (0.96; 1.82)	1.26 (0.84; 1.91)
≥ 2	1.15 (0.79; 1.69)	1.10 (0.74; 1.64)	1.88 (1.29; 2.75)	1.94 (1.19; 3.17)
No. of inappropriate medications				
0	Ref	Ref	Ref	Ref
1	1.05 (0.72; 1.53)	1.01 (0.68; 1.50)	1.22 (0.84; 1.77)	0.98 (0.61; 1.59)
≥ 2	1.12 (0.80; 1.56)	1.12 (0.79; 1.59)	1.13 (0.81; 1.59)	1.18 (0.77; 1.83)
High-risk medication^c^				
Antidiabetics	1.22 (0.91; 1.63)	1.17 (0.86; 1.60)	1.41 (1.05; 1.89)	1.10 (0.75; 1.62)
Opioids	1.74 (1.23; 2.48)	1.59 (1.11; 2.30)	2.31 (1.63; 3.28)	2.10 (1.34; 3.32)
Antibiotics	1.81 (1.14; 2.91)	1.64 (1.01; 2.68)	1.44 (0.92; 2.27)	1.77 (0.99; 3.18)
Benzodiazepines	1.38 (0.92; 2.08)	1.32 (0.87; 2.03)	1.75 (1.17; 2.61)	2.01 (1.22; 3.35)
Antidepressants	1.54 (1.13; 2.10)	1.32 (0.95; 1.83)	1.57 (1.15; 2.13)	1.45 (0.96; 2.19)
Medication complexity				
< 16.5	Ref	Ref	Ref	Ref
16.5–25.4	1.02 (0.74; 1.40)	0.95 (0.68; 1.33)	1.20 (0.87; 1.66)	0.81 (0.53; 1.22)
≥ 25.5	1.77 (1.28; 2.43)	1.53 (1.10; 2.15)	1.83 (1.33; 2.51)	1.22 (0.80; 1.86)
Adherence (MMAS-8)^© d^				
Good adherence	Ref	Ref	Ref	Ref
Medium adherence	1.01 (0.76; 1.35)	1.12 (0.83; 1.52)	0.80 (0.59; 1.06)	1.36 (0.93; 2.01)
Low adherence	0.94 (0.63; 1.38)	0.93 (0.62; 1.39)	1.13 (0.77; 1.67)	1.59 (0.95; 2.66)

Missing data: Medication adherence, EQ-VAS outcome, 47 (5.1%) and EQ-5D, 49 (5.2%), adjusted models: EQ-VAS, 46 (5.0%) and EQ-5D, 36 (4.2%); No of prescribing omissions and No of inappropriate medications, EQ-VAS outcome, 129 (13.9%) and EQ-5D, 133 (14.0%), adjusted models: EQ-VAS, 128 (14.0%) and EQ-5D, 108 (12.6%)

^a^Adjusted for DADL and smoking status

^b^Adjusted for the trial site, DADL, non-independent living, smoking status, BMI, falls in the past year and non-elective admittance

^c^Only high-risk medication (medication with a high risk for hospital (re)admissions in patients) with an association is displayed

^d^Use of Morisky medication adherence measure questionnaire is protected by US copyright laws

Due to the collinearity between medication complexity and anticholinergic and sedative burden with the number of medications, we performed a stratified analysis based on hyperpolypharmacy. The no-hyperpolypharmacy groups, with medication complexity scores ≥ 20.5 (median) and an anticholinergic and sedative score ≥ 1, were too small to analyse. In the analysis of patients with hyperpolypharmacy (EQ-VAS *n* = 503 and EQ-5D *n* = 507), an anticholinergic and sedative score of ≥ 1 and a medication complexity score ≥ 20.5 (median) were significantly associated with a lower EQ-5D score (aOR 1.82, 95% CI 1.03–3.22 and aOR: 1.93, 95% CI 1.19–3.17, respectively).

## Discussion

This study demonstrated an association between several medication use-related factors and a lower HRQoL. The use of opioids was the only factor that was associated with both lower EQ-VAS and EQ-5D. Additionally, associations between a lower EQ-VAS and hyperpolypharmacy, use of antibiotics and medication complexity were found. For lower EQ-5D, associations with anticholinergic and sedative burden, the presence of multiple prescribing omissions and benzodiazepine use were found. High medication complexity and high anticholinergic and sedative burden had a stronger association with a lower HRQoL in older patients with hyperpolypharmacy than in older patients with polypharmacy.

The median EQ-VAS (60) and EQ-5D (0.60) found were lower than the median EQ-VAS (70) and the EQ-5D (0.74) of a Dutch study with older multimorbid patients with polypharmacy in primary care [16]. The HRQoL in the Dutch study was lower than the population norms for people aged 75 years and above from the Netherlands (EQ-VAS 73 and EQ-5D 0.80) [38]. This study included hospitalised patients, potentially leading to an overall lower quality of life than in primary care. In this study population, there was higher usage of (prophylactic) antibiotics and opioids prior to hospitalisation than expected, this could be an indication that this study population was sicker than the general population. Moreover, the study population was a combination of patients from four different countries with different population norms for HRQoL [38].

Both the EQ-5D index score and the EQ-VAS were considered independent indicators of quality of life. EQ-VAS and EQ-5D measure different constructs, and therefore, associations with medication use-related factor can be different. EQ-VAS gives the self-reported overall well-being of people, whereas EQ-5D is more determined by functioning as three dimensions focus on functioning. However, the dimensions anxiety/depression and pain are also conditions that can be treated with medication. It is argued that patient experience with regard to medication is not adequately represented in the responsiveness of the EQ-5D [16]. In two cohort studies, the EQ-VAS improved after a medication optimisation intervention, but no improvement in EQ-5D index score was found [16, 17]. In this study, we observed differences in confounders between EQ-VAS and EQ-5D. Both EQ-VAS and EQ-5D were influenced by dependency in activities of daily living and smoking. However, the EQ-5D score was also influenced by nonindependent living, a high BMI, falls in the past year and nonelective admittance. These factors might affect the dimensions of mobility, self-care and usual activities and have less impact on overall well-being.

The use of opioids could be a proxy for the existence of pain as they are effective in addressing acute nociceptive pain. However, in cases of chronic pain, rapid habituation and even hyperalgesia may occur, potentially impacting quality of life negatively. The presence of neuropathic pain or side effects of opioids could be additional explanations for a lower HRQoL [27, 39, 40]. An effect was seen on both EQ-VAS and EQ-5D that can be explained by the impact pain has on overall well-being, and pain is one of the dimensions included in the EQ-5D index score.

In this study, hyperpolypharmacy, antibiotics and medication complexity were only associated with a lower EQ-VAS. For hyperpolypharmacy, earlier research regarding the association with HRQoL has produced conflicting results [5, 10–14]. Most studies have found an association between the number of medications and HRQoL, but one study did not. This study’s study population was younger, had fewer chronic conditions and used less medication, which could explain why other associations were found [5]. For antibiotics, the underlying infectious disease, a delayed effect of the antibiotics as well as side effects of antibiotics (e.g. diarrhoea) could account for the lower EQ-VAS. For medication complexity, no other studies were found that evaluated the association with HRQoL. A previous study suggested that medication complexity did not predict unplanned hospitalisations more accurately than the number of medications [9]. As the number of medications and medication complexity show collinearity, it is difficult to determine which one is the more important determinant. Therefore, we performed a stratified analysis. This analysis showed a stronger association between higher medication complexity and a lower HRQoL in patients with hyperpolypharmacy, indicating that increased intricacy of medication regimens might especially contribute to a diminished quality of life in patients with hyperpolypharmacy.

In this study, associations with anticholinergic and sedative burden, the presence of prescribing omissions and the use of benzodiazepines with lower EQ-5D index scores were found. Overtreatment, undertreatment or side effects might explain the associations found between these factors and lower EQ-5D index scores. Anticholinergic and sedative medication is often used to treat anxiety, depression or pain. The association was even stronger in older patients with hyperpolypharmacy, possibly because with increasing numbers of medications used, the number of sedative or anticholinergic medications also increases. A study among Irish community-dwelling older patients also found that high anticholinergic and sedative burden reduced QoL [24]. Other studies using different tools, like the Anticholinergic Drug Scale, similarly found that high anticholinergic burdens were associated with reduced QoL [41]. Frequent START criteria that were present in this study population were undertreatment of vitamin D [27], possibly leading to lower physical and functional well-being [42]. This might be an explanation for the association found with a lower EQ-5D. A study with the aim to develop a prognostic model for a low EQ-5D index score also found prescribing omissions prognostic factors for quality of life [5]. No associations were found between inappropriate medications and EQ-VAS and EQ-5D in this study population. The most frequent STOPP criterium present in this study population was medication without indication [27]. It is possible that even though the medication had no clear indication, patients did not experience it as a burden, and therefore no associations were found with a lower HRQoL. Benzodiazepine use is a proxy for the presence of anxiety-related problems. Benzodiazepines have limited effectiveness, and there is a risk of development of dependence with long-term usage. In older patients, there is an increased risk of side effects [43].

This study had a large study population with minimal exclusion criteria, increasing the generalisability of the results. Medical information was prospectively collected by a team of physicians and pharmacists who had full access to the patient’s medical files, resulting in a very complete dataset. Therefore, the medication use-related factors provide accurate insight into clinically relevant medication use-related factors in this population. The EQ-5D was significantly different between trial sites. The independent dimensions (like pain and anxiety/depression) also were significantly different between the trial sites (data not shown). The EQ-VAS did not differ significantly between the trial sites; this suggests that differences in EQ-5D between the countries may derive from the different experiences of the dimensions of EQ-5D. The country-specific algorithms should correctly reflect country-specific perceptions. Maybe country-specific algorithms are less suitable for this study population of relatively sick and frail patients, or maybe countries differ in EQ-5D [38].

There could be residual confounding in this study as some factors that could influence the HRQoL were not available, like data on acuteness or seriousness of the conditions, reason for the current hospital admission, income and social status. STOPP/START criteria were measured during hospitalisation. Therefore, there might be an underrepresentation of recommendations because some medications might have been stopped at hospital admission and did not generate recommendations. Moreover, there could also be a potential source of bias due to missing data, information on BMI was missing in 7.4% of patients and information on the appropriateness of medication was missing in 13.9% of patients, due to technical errors or logistic issues such as early discharge, transfer to another ward or withdrawal from the trial before the intervention [27]. Therefore, our findings concerning the influence of the appropriateness of medication should be interpreted cautiously.

While the study’s cross-sectional design limits causal inferences, the identified associations warrant attention from healthcare professionals. The results emphasize the importance of a holistic approach to medication management, where healthcare providers weigh the benefits and potential drawbacks of each medication, the underlying medical condition and their combination in light of its impact on the patient’s HRQoL. Pharmaceutical interventions have a patient- and/or problem-centred approach using patient-reported problems and tools, such as the STOPP/START criteria, to optimise prescribing. In all commonly used tools, deprescribing of anticholinergic and sedative medication is included to reduce inappropriate prescribing. However, medication complexity is not included in these tools. Furthermore, medication complexity was associated with hospitalisation, hospital readmission, and lower medication adherence, as shown in a systematic review and meta-analysis [44]. Selecting older patients with hyperpolypharmacy and high medication complexity could, therefore, be an approach to identifying patients who could benefit from a pharmaceutical intervention. Further studies should assess whether a reduction of medication complexity impacts patients’ quality of life. Additionally, further longitudinal research is warranted to establish causal relationships and explore the long-term effects of medication use on HRQoL in this population. To fully understand the association between medication complexity and HRQoL, further research is necessary, including patients’ perspectives and experiences, to develop strategies to optimize medication management and assess the effects of the implications of those strategies.

In conclusion, an association was found between several medication use-related factors and a lower HRQoL in multimorbid older hospitalised patients with polypharmacy. It is crucial for healthcare professionals to actively address quality of life in older patients taking multiple medications, especially in those with high medication complexity, high anticholinergic and sedative burden, presence of multiple prescribing omissions and/or using opioids, benzodiazepines or antibiotics. Other studies have also identified these medication use-related factors as being associated with low HRQoL. However, medication complexity is a novel factor, which should be considered when evaluating medication use of older patients with (hyper)polypharmacy.

## Data Availability

Data for this study will be made available to others in the scientific community upon request after publication. Data will be made available for scientific purposes for researchers whose proposed use of the data has been approved by the OPERAM publication committee.
