# Correction: The association between medication use and health-related quality of life in multimorbid older patients with polypharmacy

**DOI:** 10.1007/s41999-024-01072-0

**Published:** 2024-10-25

**Authors:** Charlotte Falke, Fatma Karapinar, Marcel Bouvy, Mariëlle Emmelot, Svetlana Belitser, Benoit Boland, Denis O’Mahony, Kevin D. Murphy, Moa Haller, Paola Salari, Matthias Schwenkglenks, Nicolas Rodondi, Toine Egberts, Wilma Knol

**Affiliations:** 1 Division of Pharmacoepidemiology and Clinical Pharmacology, Faculty of Science, Utrecht Institute for Pharmaceutical Sciences, Utrecht University, Utrecht, the Netherlands; 2 Department of Clinical Pharmacy and Toxicology, Maastricht University Medical Center+, Maastricht, the Netherlands; 3 Department of Clinical Pharmacy, CARIM Cardiovascular Research Institute Maastricht, Maastricht University, Maastricht, the Netherlands; 4 Geriatric Medicine Department and Expertise Centre Pharmacotherapy in Old Persons, University Medical Centre Utrecht, Utrecht University Utrecht, Utrecht, the Netherlands; 5 Institute of Health and Society (IRSS), Université Catholique de Louvain (UCLouvain), Ottignies-Louvain-La-Neuve, Belgium; 6 Geriatric Medicine, Cliniques Universitaires Saint-Luc, avenue Hippocrate 10, 1200 Brussels, Belgium; 7 Department of Medicine, University College Cork, Cork, Ireland; 8 Department of Geriatric and Stroke Medicine, Cork University Hospital, Cork, Ireland; 9 School of Pharmacy, University College Cork, Cork, Ireland; 10 Institute of Primary Health Care (BIHAM), University of Bern, Bern, Switzerland; 11 Department of General Internal Medicine, Inselspital, Bern University Hospital, University of Bern, Bern, Switzerland; 12 Institute of Pharmaceutical Medicine (ECPM), University of Basel, Klingelbergstrasse, 61, 4056 Basel, Switzerland; 13 Present Address: Joint Research Centre (JRC), European Commission, Ispra, Italy

**Correction: European Geriatric Medicine (2024)** 10.1007/s41999-024-01036-4

In Tables 2 and 3 of this article, the data in the row headed “Number of patients” was mistakenly listed in the header of the tables.

The correct tables should have shown as follows:

**Table:** **Table 2** Characteristics of the patients divided into groups based on HRQoL^a^

	Lower EQ-VAS	Higher EQ-VAS	*p*-value	Lower EQ-5D	Higher EQ-5D	*p*-value
Total number of patients	449	478		402	548	
Age ≥ 80 years	199 (44.3)	220 (46.0)	0.649	197 (49.0)	236 (43.1)	0.080
Female	224 (49.9)	210 (43.9)	0.080	205 (51.0)	232 (42.3)	0.010
Body mass index ≥ 30 kg/m^2^	94 (22.7)	99 (22.1)	0.909	99 (26.3)	97 (19.2)	0.016
Current smoker	46 (10.2)	30 (6.3)	0.038	43 (10.7)	32 (5.9)	0.009
Level of education			0.026			0.025
Less than high school	140 (31.5)	113 (23.8)		127 (32.0)	131 (24.1)	
High school	219 (49.3)	253 (53.3)		194 (48.9)	288 (53.0)	
University	85 (19.1)	109 (22.9)		76 (19.1)	124 (22.8)	
Nonindependent living	99 (22.0)	62 (13.0)	< 0.001	114 (28.4)	53 (9.7)	< 0.001
Dementia	37 (8.2)	25 (5.2)	0.089	31 (7.7)	34 (6.2)	0.436
Renal impairment (eGFR < 50 ml/min)	156 (35.9)	151 (32.1)	0.254	132 (34.3)	174 (32.2)	0.544
DADL (Barthel index) ^b^: Moderate or severe	269 (60.7)	164 (34.6)	< 0.001	276 (70.2)	169 (31.0)	< 0.001
≥ 1 Fall(s) during past year	99 (22.2)	77 (16.2)	0.025	103 (25.9)	77 (14.2)	< 0.001
Trial site			0.589			< 0.001
Bern	200 (44.5)	228 (47.7)		129 (32.1)	309 (56.4)	
Cork	63 (14.0)	71 (14.9)		40 (10.0)	98 (17.9)	
Louvain	71 (15.8)	74 (15.5)		68 (16.9)	80 (14.6)	
Utrecht	115 (25.6)	105 (22.0)		165 (41.0)	61 (11.1)	
Ward specialism (surgical/medical): Surgical	88 (19.6)	103 (21.5)	0.514	95 (23.6)	102 (18.6)	0.071
Type of hospital admittance: Nonelective	335 (75.3)	364 (76.3)	0.774	285 (72.0)	430 (78.5)	0.026
≥ 1 Hospital admission(s) during past year	112 (25.0)	96 (20.1)	0.090	101 (25.2)	110 (20.1)	0.075
Medication use-related factors						
Hyperpolypharmacy (≥ 10 medications)	264 (58.8)	239 (50.0)	0.009	236 (58.7)	271 (49.5)	0.006
Anticholinergic and sedative burden			0.834			< 0.001
DBI = 0	221 (49.2)	244 (51.0)		178 (44.3)	303 (55.3)	
DBI 0–1	134 (29.8)	135 (28.2)		119 (29.6)	154 (28.1)	
DBI ≥ 1	94 (20.9)	99 (20.7)		105 (26.1)	91 (16.6)	
Appropriateness of medication:						
No. of prescribing omissions			0.463			0.004
0	186 (49.5)	227 (53.8)		160 (45.5)	259 (55.7)	
1	121 (32.2)	122 (28.9)		113 (32.1)	138 (29.7)	
≥ 2	69 (18.4)	73 (17.3)		79 (22.4)	68 (14.6)	
No. of inappropriate medications			0.798			0.582
0–1	102 (27.1)	122 (28.9)		93 (26.4)	137 (29.5)	
2–4	100 (26.6)	114 (27.0)		99 (28.1)	120 (25.8)	
≥ 5	174 (46.3)	186 (44.1)		160 (45.5)	208 (44.7)	
High-risk medication^c^						
Antidiabetics	126 (28.1)	116 (24.3)	0.215	118 (29.4)	125 (22.8)	0.027
Opioids	94 (20.9)	63 (13.2)	0.002	94 (23.4)	64 (11.7)	< 0.001
Antibiotics	50 (11.1)	31 (6.5)	0.017	42 (10.4)	41 (7.5)	0.138
Benzodiazepines	60 (13.4)	48 (10.0)	0.141	60 (14.9)	50 (9.1)	0.008
Antidepressants	118 (26.3)	90 (18.8)	0.008	107 (26.6)	103 (18.8)	0.005
Medication complexity			< 0.001			< 0.001
< 16.5	130 (29.0)	169 (35.4)		114 (28.4)	203 (37.0)	
16.5–25.4	137 (30.5)	175 (36.6)		127 (31.6)	188 (34.3)	
≥ 25.5	182 (40.5)	134 (28.0)		161 (40.0)	157 (28.6)	
Medication adherence (MMAS-8)^© d^			0.930			0.139
Low adherence	64 (15.1)	73 (16.0)		65 (17.2)	74 (14.1)	
Medium adherence	173 (40.8)	183 (40.1)		139 (36.8)	225 (43.0)	
Good adherence	187 (44.1)	200 (43.9)		174 (46.0)	224 (42.8)	

Missing data: EQ-5D, 5 (0.5%); EQ-VAS, 28 (2.9%); BMI, 71 (7.4%); smoking status, 1 (0.1%); number of falls during the previous year, 8 (0.8%); level of education, 10 (1.0%); number of hospitalisations in the previous year, 2 (0.2%); admission type, 6 (0.6%); renal function, 24 (2.5%); Barthel Index of ADL, 11 (1.2%); medication adherence, 49 (5.1%); No of prescribing omissions, 133 (13.9%); No of inappropriate medications, 133 (13.9%)

^a^The values are numbers (percentages)

^b^Dependency on activities of daily living (DADL) measured with the Barthel index, a score of ≤ 60 is considered a severe dependency, 60–90 is considered moderate dependency and > 90 almost no dependency[10]

^c^Only the high-risk medication (medication with a high risk for hospital (re)admissions in patients) with significant differences in proportions are displayed

^d^Use of the Morisky Medication Adherence Measure questionnaire is protected by U.S. copyright laws. Permission for use is required. A license agreement was obtained from Donald E Morisky, ScD, ScM, MSPH, Professor, Department of Community Health Sciences, UCLA Fielding School of Public Health, 650 Charles E Young Drive South, Los Angeles, CA 90095–1772, USA (dmorisky@ucla.edu)

**Table:** **Table 3** Association of medication use-related factors with lower EQ-VAS and EQ-5D index scores

Medication use-related factors	crude OR (CI) EQ-VAS	aOR (CI) EQ-VAS ^a^	crude OR (CI) EQ-5D	aOR (CI) EQ-5D ^b^
Total number of patients	927	916	950	855
Hyperpolypharmacy	1.43 (1.10; 1.85)	1.37 (1.05; 1.80)	1.45 (1.12; 1.89)	1.30 (0.93; 1.84)
Anticholinergic and sedative burden				
DBI = 0	Ref	Ref	Ref	Ref
DBI 0–1	1.10 (0.81; 1.48)	1.05 (0.76; 1.43)	1.32 (0.97; 1.78)	1.11 (0.75; 1.64)
DBI ≥ 1	1.05 (0.75; 1.47)	0.88 (0.62; 1.25)	1.96 (1.40; 2.75)	1.73 (1.11; 2.69)
Appropriateness of medication				
No. of prescribing omissions				
0	Ref	Ref	Ref	Ref
1	1.21 (0.88; 1.66)	1.16 (0.83; 1.62)	1.33 (0.96; 1.82)	1.26 (0.84; 1.91)
≥ 2	1.15 (0.79; 1.69)	1.10 (0.74; 1.64)	1.88 (1.29; 2.75)	1.94 (1.19; 3.17)
No. of inappropriate medications				
0	Ref	Ref	Ref	Ref
1	1.05 (0.72; 1.53)	1.01 (0.68; 1.50)	1.22 (0.84; 1.77)	0.98 (0.61; 1.59)
≥ 2	1.12 (0.80; 1.56)	1.12 (0.79; 1.59)	1.13 (0.81; 1.59)	1.18 (0.77; 1.83)
High-risk medication^c^				
Antidiabetics	1.22 (0.91; 1.63)	1.17 (0.86; 1.60)	1.41 (1.05; 1.89)	1.10 (0.75; 1.62)
Opioids	1.74 (1.23; 2.48)	1.59 (1.11; 2.30)	2.31 (1.63; 3.28)	2.10 (1.34; 3.32)
Antibiotics	1.81 (1.14; 2.91)	1.64 (1.01; 2.68)	1.44 (0.92; 2.27)	1.77 (0.99; 3.18)
Benzodiazepines	1.38 (0.92; 2.08)	1.32 (0.87; 2.03)	1.75 (1.17; 2.61)	2.01 (1.22; 3.35)
Antidepressants	1.54 (1.13; 2.10)	1.32 (0.95; 1.83)	1.57 (1.15; 2.13)	1.45 (0.96; 2.19)
Medication complexity				
< 16.5	Ref	Ref	Ref	Ref
16.5–25.4	1.02 (0.74; 1.40)	0.95 (0.68; 1.33)	1.20 (0.87; 1.66)	0.81 (0.53; 1.22)
≥ 25.5	1.77 (1.28; 2.43)	1.53 (1.10; 2.15)	1.83 (1.33; 2.51)	1.22 (0.80; 1.86)
Adherence (MMAS-8)^© d^				
Good adherence	Ref	Ref	Ref	Ref
Medium adherence	1.01 (0.76; 1.35)	1.12 (0.83; 1.52)	0.80 (0.59; 1.06)	1.36 (0.93; 2.01)
Low adherence	0.94 (0.63; 1.38)	0.93 (0.62; 1.39)	1.13 (0.77; 1.67)	1.59 (0.95; 2.66)

Missing data: Medication adherence, EQ-VAS outcome, 47 (5.1%) and EQ-5D, 49 (5.2%), adjusted models: EQ-VAS, 46 (5.0%) and EQ-5D, 36 (4.2%); No of prescribing omissions and No of inappropriate medications, EQ-VAS outcome, 129 (13.9%) and EQ-5D, 133 (14.0%), adjusted models: EQ-VAS, 128 (14.0%) and EQ-5D, 108 (12.6%)

^a^Adjusted for DADL and smoking status

^b^Adjusted for the trial site, DADL, non-independent living, smoking status, BMI, falls in the past year and non-elective admittance

^c^Only high-risk medication (medication with a high risk for hospital (re)admissions in patients) with an association is displayed

^d^Use of Morisky medication adherence measure questionnaire is protected by US copyright laws

The incorrect tables showed as follows:

**Table:** **Table 2** Characteristics of the patients divided into groups based on HRQoL^a^

Total number of patients	Lower EQ-VAS	Higher EQ-VAS	*p*-value	Lower EQ-5D	Higher EQ-5D	*p*-value
	449	478		402	548	
Age ≥ 80 years	199 (44.3)	220 (46.0)	0.649	197 (49.0)	236 (43.1)	0.080
Female	224 (49.9)	210 (43.9)	0.080	205 (51.0)	232 (42.3)	0.010
Body mass index ≥ 30 kg/m^2^	94 (22.7)	99 (22.1)	0.909	99 (26.3)	97 (19.2)	0.016
Current smoker	46 (10.2)	30 (6.3)	0.038	43 (10.7)	32 (5.9)	0.009
Level of education			0.026			0.025
Less than high school	140 (31.5)	113 (23.8)		127 (32.0)	131 (24.1)	
High school	219 (49.3)	253 (53.3)		194 (48.9)	288 (53.0)	
University	85 (19.1)	109 (22.9)		76 (19.1)	124 (22.8)	
Nonindependent living	99 (22.0)	62 (13.0)	< 0.001	114 (28.4)	53 (9.7)	< 0.001
Dementia	37 (8.2)	25 (5.2)	0.089	31 (7.7)	34 (6.2)	0.436
Renal impairment (eGFR < 50 ml/min)	156 (35.9)	151 (32.1)	0.254	132 (34.3)	174 (32.2)	0.544
DADL (Barthel index) ^b^: Moderate or severe	269 (60.7)	164 (34.6)	< 0.001	276 (70.2)	169 (31.0)	< 0.001
≥ 1 Fall(s) during past year	99 (22.2)	77 (16.2)	0.025	103 (25.9)	77 (14.2)	< 0.001
Trial site			0.589			< 0.001
Bern	200 (44.5)	228 (47.7)		129 (32.1)	309 (56.4)	
Cork	63 (14.0)	71 (14.9)		40 (10.0)	98 (17.9)	
Louvain	71 (15.8)	74 (15.5)		68 (16.9)	80 (14.6)	
Utrecht	115 (25.6)	105 (22.0)		165 (41.0)	61 (11.1)	
Ward specialism (surgical/medical): Surgical	88 (19.6)	103 (21.5)	0.514	95 (23.6)	102 (18.6)	0.071
Type of hospital admittance: Nonelective	335 (75.3)	364 (76.3)	0.774	285 (72.0)	430 (78.5)	0.026
≥ 1 Hospital admission(s) during past year	112 (25.0)	96 (20.1)	0.090	101 (25.2)	110 (20.1)	0.075
Medication use-related factors						
Hyperpolypharmacy (≥ 10 medications)	264 (58.8)	239 (50.0)	0.009	236 (58.7)	271 (49.5)	0.006
Anticholinergic and sedative burden			0.834			< 0.001
DBI = 0	221 (49.2)	244 (51.0)		178 (44.3)	303 (55.3)	
DBI 0–1	134 (29.8)	135 (28.2)		119 (29.6)	154 (28.1)	
DBI ≥ 1	94 (20.9)	99 (20.7)		105 (26.1)	91 (16.6)	
Appropriateness of medication:						
No. of prescribing omissions			0.463			0.004
0	186 (49.5)	227 (53.8)		160 (45.5)	259 (55.7)	
1	121 (32.2)	122 (28.9)		113 (32.1)	138 (29.7)	
≥ 2	69 (18.4)	73 (17.3)		79 (22.4)	68 (14.6)	
No. of inappropriate medications			0.798			0.582
0–1	102 (27.1)	122 (28.9)		93 (26.4)	137 (29.5)	
2–4	100 (26.6)	114 (27.0)		99 (28.1)	120 (25.8)	
≥ 5	174 (46.3)	186 (44.1)		160 (45.5)	208 (44.7)	
High-risk medication^c^						
Antidiabetics	126 (28.1)	116 (24.3)	0.215	118 (29.4)	125 (22.8)	0.027
Opioids	94 (20.9)	63 (13.2)	0.002	94 (23.4)	64 (11.7)	< 0.001
Antibiotics	50 (11.1)	31 (6.5)	0.017	42 (10.4)	41 (7.5)	0.138
Benzodiazepines	60 (13.4)	48 (10.0)	0.141	60 (14.9)	50 (9.1)	0.008
Antidepressants	118 (26.3)	90 (18.8)	0.008	107 (26.6)	103 (18.8)	0.005
Medication complexity			< 0.001			< 0.001
< 16.5	130 (29.0)	169 (35.4)		114 (28.4)	203 (37.0)	
16.5–25.4	137 (30.5)	175 (36.6)		127 (31.6)	188 (34.3)	
≥ 25.5	182 (40.5)	134 (28.0)		161 (40.0)	157 (28.6)	
Medication adherence (MMAS-8)^© d^			0.930			0.139
Low adherence	64 (15.1)	73 (16.0)		65 (17.2)	74 (14.1)	
Medium adherence	173 (40.8)	183 (40.1)		139 (36.8)	225 (43.0)	
Good adherence	187 (44.1)	200 (43.9)		174 (46.0)	224 (42.8)	

Missing data: EQ-5D, 5 (0.5%); EQ-VAS, 28 (2.9%); BMI, 71 (7.4%); smoking status, 1 (0.1%); number of falls during the previous year, 8 (0.8%); level of education, 10 (1.0%); number of hospitalisations in the previous year, 2 (0.2%); admission type, 6 (0.6%); renal function, 24 (2.5%); Barthel Index of ADL, 11 (1.2%); medication adherence, 49 (5.1%); No of prescribing omissions, 133 (13.9%); No of inappropriate medications, 133 (13.9%)

^a^The values are numbers (percentages)

^b^Dependency on activities of daily living (DADL) measured with the Barthel index, a score of ≤ 60 is considered a severe dependency, 60–90 is considered moderate dependency and > 90 almost no dependency[10]

^c^Only the high-risk medication (medication with a high risk for hospital (re)admissions in patients) with significant differences in proportions are displayed

^d^Use of the Morisky Medication Adherence Measure questionnaire is protected by U.S. copyright laws. Permission for use is required. A license agreement was obtained from Donald E Morisky, ScD, ScM, MSPH, Professor, Department of Community Health Sciences, UCLA Fielding School of Public Health, 650 Charles E Young Drive South, Los Angeles, CA 90095–1772, USA (dmorisky@ucla.edu)

**Table:** **Table 3** Association of medication use-related factors with lower EQ-VAS and EQ-5D index scores

Medication use-related factors	crude OR (CI) EQ-VAS	aOR (CI) EQ-VAS ^a^	crude OR (CI) EQ-5D	aOR (CI) EQ-5D ^b^
Total number of patients	927	916	950	855
Hyperpolypharmacy	1.43 (1.10; 1.85)	1.37 (1.05; 1.80)	1.45 (1.12; 1.89)	1.30 (0.93; 1.84)
Anticholinergic and sedative burden				
DBI = 0	Ref	Ref	Ref	Ref
DBI 0–1	1.10 (0.81; 1.48)	1.05 (0.76; 1.43)	1.32 (0.97; 1.78)	1.11 (0.75; 1.64)
DBI ≥ 1	1.05 (0.75; 1.47)	0.88 (0.62; 1.25)	1.96 (1.40; 2.75)	1.73 (1.11; 2.69)
Appropriateness of medication				
No. of prescribing omissions				
0	Ref	Ref	Ref	Ref
1	1.21 (0.88; 1.66)	1.16 (0.83; 1.62)	1.33 (0.96; 1.82)	1.26 (0.84; 1.91)
≥ 2	1.15 (0.79; 1.69)	1.10 (0.74; 1.64)	1.88 (1.29; 2.75)	1.94 (1.19; 3.17)
No. of inappropriate medications				
0	Ref	Ref	Ref	Ref
1	1.05 (0.72; 1.53)	1.01 (0.68; 1.50)	1.22 (0.84; 1.77)	0.98 (0.61; 1.59)
≥ 2	1.12 (0.80; 1.56)	1.12 (0.79; 1.59)	1.13 (0.81; 1.59)	1.18 (0.77; 1.83)
High-risk medication^c^				
Antidiabetics	1.22 (0.91; 1.63)	1.17 (0.86; 1.60)	1.41 (1.05; 1.89)	1.10 (0.75; 1.62)
Opioids	1.74 (1.23; 2.48)	1.59 (1.11; 2.30)	2.31 (1.63; 3.28)	2.10 (1.34; 3.32)
Antibiotics	1.81 (1.14; 2.91)	1.64 (1.01; 2.68)	1.44 (0.92; 2.27)	1.77 (0.99; 3.18)
Benzodiazepines	1.38 (0.92; 2.08)	1.32 (0.87; 2.03)	1.75 (1.17; 2.61)	2.01 (1.22; 3.35)
Antidepressants	1.54 (1.13; 2.10)	1.32 (0.95; 1.83)	1.57 (1.15; 2.13)	1.45 (0.96; 2.19)
Medication complexity				
< 16.5	Ref	Ref	Ref	Ref
16.5–25.4	1.02 (0.74; 1.40)	0.95 (0.68; 1.33)	1.20 (0.87; 1.66)	0.81 (0.53; 1.22)
≥ 25.5	1.77 (1.28; 2.43)	1.53 (1.10; 2.15)	1.83 (1.33; 2.51)	1.22 (0.80; 1.86)
Adherence (MMAS-8)^© d^				
Good adherence	Ref	Ref	Ref	Ref
Medium adherence	1.01 (0.76; 1.35)	1.12 (0.83; 1.52)	0.80 (0.59; 1.06)	1.36 (0.93; 2.01)
Low adherence	0.94 (0.63; 1.38)	0.93 (0.62; 1.39)	1.13 (0.77; 1.67)	1.59 (0.95; 2.66)

Missing data: Medication adherence, EQ-VAS outcome, 47 (5.1%) and EQ-5D, 49 (5.2%), adjusted models: EQ-VAS, 46 (5.0%) and EQ-5D, 36 (4.2%); No of prescribing omissions and No of inappropriate medications, EQ-VAS outcome, 129 (13.9%) and EQ-5D, 133 (14.0%), adjusted models: EQ-VAS, 128 (14.0%) and EQ-5D, 108 (12.6%)

^a^Adjusted for DADL and smoking status

^b^Adjusted for the trial site, DADL, non-independent living, smoking status, BMI, falls in the past year and non-elective admittance

^c^Only high-risk medication (medication with a high risk for hospital (re)admissions in patients) with an association is displayed

^d^Use of Morisky medication adherence measure questionnaire is protected by US copyright laws

The original article has been corrected.

